# Functional Characterization of PknI-Rv2159c Interaction in Redox Homeostasis of *Mycobacterium tuberculosis*

**DOI:** 10.3389/fmicb.2016.01654

**Published:** 2016-10-21

**Authors:** Arunkumar Venkatesan, Kannan Palaniyandi, Divakar Sharma, Deepa Bisht, Sujatha Narayanan

**Affiliations:** ^1^Department of Immunology, National Institute for Research in TuberculosisChennai, India; ^2^Department of Biochemistry, National JALMA Institute for Leprosy and other Mycobacterial DiseasesAgra, India

**Keywords:** PknI, Rv2159c, antisense knockdown, alkylhydroperoxidase, protein-protein interactions, 2D gel electrophoresis

## Abstract

*Mycobacterium tuberculosis* adapts to stress conditions by responding to the signals from its external environment. *M. tuberculosis* genome encodes 11 eukaryotic like serine/threonine protein kinases (STPK) and their importance in regulating the physiology and virulence of the bacteria are being explored. Previous study from our lab identified the *M. tuberculosis* STPK, PknI interacts with two peroxidase proteins such as Rv2159c and Rv0148. In this study, we have characterized the biological function behind the PknI-Rv2159c interaction in *M. tuberculosis*. Point mutation of Ala-Gly-Trp motif identified that only Ala49 and Gly50 amino acids of Rv2159c are responsible for interaction and there is no phosphorylation involved in the PknI-Rv2159c interaction. Rv2159c is a member from the carboxymuconolactone decarboxylase family with peroxidase activity. Enzymatic assays with catalytic site point mutants showed that Cys84 of Rv2159c was responsible for its alkylhydroperoxidase activity. Interestingly, interaction with PknI increased its peroxidase activity by several folds. Gene knockdown of Rv2159c in *M. tuberculosis* showed increased sensitivity to peroxides such as cumene hydroperoxide and hydrogen peroxide. Proteomic analysis of differentially expressing Rv2159c strains by 2D gel electrophoresis and mass spectrometry revealed the differential abundance of 21 proteins. The total absence of oxidoreductase, GuaB1 suggests the essential role of Rv2159c in redox maintenance. Our findings provide new insights on signaling mechanisms of PknI in maintaining the redox homeostasis during oxidative stresses.

## Introduction

Tuberculosis (TB) is an infectious disease, caused by one of the most successful human pathogen, *Mycobacterium tuberculosis*. World health organization (WHO) estimated about 1.5 million TB deaths and 9 million new TB cases in 2013 (World Health Organisation, 2014). Furthermore, the increased emergence of multidrug-resistant (MDR), extensively drug-resistant (XDR) TB and HIV-TB co-infection remains a global health concern (Kaufmann and Parida, 2007).

Proteins do not act isolated inside the living cells, rather they perform their functions by interacting with respective substrates. In *M. tuberculosis*, the signal transduction is largely controlled by eukaryotic like STPKs, two-component signal transduction systems (TCS) and extra cytoplasmic function sigma factors (Bretl et al., 2011). The presence of equal numbers of STPKs and TCSs suggests the complex signaling mechanism of the bacteria (Av-Gay and Everett, 2000). PknI is one of the eleven STPKs in *M. tuberculosis* was shown to be important for balanced growth during acidic pH and low oxygen environments. In addition, loss of *pknI* leads to hypervirulence in both *in vitro* and *in vivo* infection models (Gopalaswamy et al., 2009). Previous study from our lab identified the physical interaction between STPK, PknI and Rv2159c. The study also suggest Ala-Gly-Trp motif of Rv2159c is responsible for PknI interaction (Venkatesan et al., 2015).

Inside the host, *M. tuberculosis* preferentially replicates in the macrophages and later they recruit lymphocytes and dendritic cells to form granulomas. During the course of infection, *M. tuberculosis* is exposed to reactive oxygen species (ROS) and reactive nitrogen species (RNS) generated by the host immune system (Macmicking et al., 1997; Cooper et al., 2000). To counteract these stresses, *M. tuberculosis* uses a variety of antioxidant defense mechanisms before they damage the cell organelles. *oxyR* and *soxR* are the two major global regulators that regulate the prokaryotes against peroxides and superoxides respectively. Despite the loss of functional *oxyR* in *M. tuberculosis*, the peroxide stress is managed by its close homolog *ahpC*, an alkylhydroperoxide reductase that detoxifies the organic peroxides (Deretic et al., 1995; Guimaraes et al., 2005). *M. tuberculosis* strains resistant to isoniazid drug were observed to be devoid of peroxidase activity. However, the mutations associated with *katG* were compensated by increased expression of *ahpC* and thereby controlling the oxidative and nitrosative stress conditions (Sherman et al., 1996). Additionally, its non-homolog protein AhpD, a low reactive alkylhydroperoxidase of *M. tuberculosis* also helps in reducing the disulfide bridge formation between the cysteine amino acids (Hillas et al., 2000).

Rv2159c is one of the conserved hypothetical proteins of *M. tuberculosis*. The closely related orthologs suggested that Rv2159c belongs to the family of alkylhydroperoxidases. It also shares its sequence homology with other mycobacterial members such as *M. bovis, M. africanum, M. caprae, M. canetti*, and *M. orygis*. The protein has a carboxymucanolactone decarboxylase (CMD) domain in its structure and appears to contain two catalytic site cysteine residues Cys-Pro-Try-Cys, responsible for peroxidase activity. Rv2159c is present adjacent to the *dcw* operon in the genome and it forms a separate operon with two uncharacterized genes such as Rv2160c and Rv2161c (Munshi et al., 2013). The expression of Rv2159c was identified to specifically increase only in the virulent *M. tuberculosis* than in the avirulent *M. tuberculosis* H37Ra and *M. bovis* (Golby et al., 2007; Li et al., 2010). However, the molecular function of alkylhydroperoxidase, Rv2159c remains uncharacterized.

In this study, we report the cloning, expression, purification and catalytic characterization of Rv2159c variants. Furthermore, the *in vitro* protein interaction studies showed the PknI interacts with Rv2159c through phosphorylation independent manner and the Ala49 and Gly50 amino acids of Rv2159c is needed for the interaction. In addition, we also investigated the functional role of Rv2159c in *M. tuberculosis* by using an antisense RNA approach. The Rv2159c gene knockdown strain showed increased *in vitro* growth rate and decreased resistance to peroxide mediated killing. Proteomic approaches identified the probable interacting proteins for Rv2159c. The results suggested that PknI activates Rv2159c during oxidative stress conditions of the bacteria and thereby maintains the cellular homeostasis.

## Materials and methods

### Plasmids, bacterial strains and media

All plasmids and constructs are listed in Table S1 and oligonucleotides are listed in Table S2. The *Escherichia coli* strains DH5α and BL21 (DE3) (Invitrogen) were used for cloning and expression of recombinant proteins. *E. coli* cells were grown in luria bertani (LB) medium with constant shaking at 37°C. *M. tuberculosis* H37Rv strain was grown in Middlebrook 7H9 broth (Difco) supplemented with 10% oleic acid-albumin-dextrose-catalase (OADC), 0.2% glycerol and 0.05% Tween 80 or 7H10 agar (Difco) supplemented with OADC at 37°C. The following antibiotics were supplemented: ampicillin at 50 μg/ml for *E. coli* and kanamycin (40 μg/ml for *E. coli* and 20 μg/ml for *M. tuberculosis*) whenever needed.

### Cloning, overexpression and purification of Rv2159c

*M. tuberculosis* genomic DNA was isolated using CTAB-NaCl method as described previously (Baess, 1974). The coding region of Rv2159c from *M. tuberculosis* was amplified by PCR using respective oligonucleotide primers (Table S2). PCR amplified Rv2159c gene was further cloned into pGEX 4T-1 vector harboring *tac* promoter. The resultant clone was confirmed through restriction digestion and DNA sequencing.

For recombinant protein expression, the pDVA 2159-GST clone was transformed into *E. coli* BL21 (DE3) cells and protein synthesis was induced with 0.5 mM isopropyl β-D-1-thiogalactopyranoside (IPTG). The induced cells were grown for 4 h at 37°C and cells were harvested by centrifugation at 6000 × g for 10 min. The cell pellet was re-suspended in buffer A (50 mM Tris chloride pH: 8, 300 mM NaCl, 20% Glycerol, and 0.5 mM Phenyl methyl sulfonyl fluoride) and sonicated at 2.5 Hz on ice using ultrasonic homogenizer FS-600N (6 cycles; 30 s pulse and 1 min rest). The lysate was centrifuged at 15,000 × g for 15 min at 4°C. The crude lysate containing proteins was bound to glutathione sepharose resin (Amersham) and incubated at 8°C in end-to-end shaker for 2 h. Column was washed with 8 column volumes of buffer A and the recombinant GST tagged Rv2159c protein was eluted with buffer A containing 20 mM of reduced glutathione. The resultant fractions were dialyzed against 0.5X buffer A, concentrated and stored at −80°C until further use.

### Site directed mutagenesis

The active and catalytic sites of Rv2159c were mutated using QuickChange II XL site directed mutagenesis (SDM) kit (Stratagene, Germany) as described by the manufacturer. The pDVA 2159-GST plasmid was used as a template to create the isogenic forms. Mutagenic primers were designed to modify the respective amino acids such as A49P, G50A, W51A, C81S, and C84S. Combinations of mutations were also generated at A49/50/51P and C81/84S. The PCR amplified mutated DNAs were digested with *DpnI* and further transformed into *E. coli* BL21 (DE3) cells. All the mutations were verified with DNA sequencing.

### Pull down assay

Affinity pull-down assay was performed in Poly-Prep chromatography columns (Bio-Rad, USA) with Ni-Nitrilotriacetic acid (Ni-NTA) resin (Invitrogen, USA) and glutathione sepharose resin (Amersham) respectively. The recombinant His-PknI was over-expressed and purified as mentioned (Gopalaswamy et al., 2004). The nickel affinity pull down was performed by incubating the cytoplasmic crude lysate containing recombinant His-PknI protein with Ni-NTA resin for 2 h at 4°C under gentle agitation. The columns were washed with buffer A containing 40 mM imidazole and incubated with the cytoplasmic crude lysate containing GST-Rv2159c recombinant protein for 4 h at 4°C under gentle agitation. Unbound proteins were removed by washing the column with 10 column volumes of buffer A. The interacting proteins were eluted with buffer A containing 200 mM imidazole. In the same way, glutathione affinity pull down assay was also performed by incubating the crude lysates containing recombinant GST-Rv2159c and His-PknI proteins with glutathione sepharose resin and eluted using buffer A containing 20 mM reduced glutathione. Eluted proteins were separated by SDS-PAGE and subjected to far-western blotting (FWB).

### Far-western blotting

The pull down assay eluted interacting proteins was separated on a 10% SDS-PAGE gel, transferred to polyvinylidene flouride (PVDF) membrane and subjected to FWB as mentioned with slight modifications (Wu et al., 2007). The proteins in the membrane was refolded by incubation with 6 M, 3 M, 1 M, and then 0.1 M Guanidine-HCl in AC buffer (5 M NaCl, 1 M Tris, pH:7.5, 0.5 M EDTA, 10% Tween-20, 2% Skim milk powder, 10% Glycerol, and 1 M DTT) for 30 min at room temperature (RT). Then, the blot was completely renatured with AC buffer and left overnight at 4°C. The membrane was then blocked with 5% skimmed milk and incubated with purified bait proteins, His-PknI and GST-Rv2159c respectively for 6–7 h at 4°C. Further, the membrane was probed with anti-His and anti-GST antibodies respectively and visualized using enhanced chemiluminescent (ECL) detection. The intensity of protein bands was also quantified using densitometer scanning and Quantity One software (Bio-Rad, USA). Finally, the percentage difference in expression was quantified using the formula, ((V_1_ − V_2_)/((V_1_ + V_2_)/2)) ^*^ 100.

### *In vitro* kinase assay

Phosphorylation reactions were carried out using [γ-^32^P] ATP (BRIT, Hyderabad) as the phosphate donor in kinase buffer (20 mM Tris-HCl, pH 7.5, 5 mM MgCl2, 1 mM DTT). Briefly, the PknI and Rv2159c proteins were incubated with kinase buffer in the presence of 5 μCi of [γ-^32^P] ATP for 30 min at 25°C. The reactions were stopped by the addition of SDS-PAGE sample buffer and heated at 95°C for 5 min. The samples were separated by SDS-PAGE, gels were dried and analyzed by autoradiography. Control reactions were also performed in kinase buffer containing [γ-^32^P] ATP without proteins or with non-specific protein.

### HPLC analysis for enzyme activity of Rv2159c

The Rv2159c, Rv2159c_C81S, Rv2159c_C84S, Rv2159c_C81/84S, PknI-Rv2159c complex proteins and the substrate, cumene hydroperoxide (CHP) (Sigma) in equal concentration (5 μM) were equilibrated in KPi buffer (50 mM KPi, pH 7.0, 100 mM KCl, 0.1 mM EDTA, and 5% Glycerol) for 120 min. The reaction was then stopped by addition of an equal volume of 6% acetic acid in acetonitrile. Reaction mixture was centrifuged and the supernatant was injected onto a Hewlett-Packard 1090 HPLC system equipped with an Axxiom ODS (4.6 × 250 mm) reverse phase HPLC column. The detector was set at 260 nm and the products were separated using 20% acetonitrile and 80% water at a flow rate of 1 ml/min. Control reactions were also performed in same KPi buffer without the enzyme or with a non-specific protein Ffh or with PknI protein alone. The decomposed product, Acetophenone was measured along with acetophenone analytical standard (Sigma) under identical elution conditions.

### DTT-dependent activity assay

The rate of dithiotheritol (DTT) oxidation catalyzed by WT and catalytic site mutants of Rv2159c in the presence of peroxide substrate (*tert*-butyl hydroperoxide) was measured by absorbance at 310 nm. The WT and catalytic site mutants (Rv2159c_C81S, Rv2159c_C84S, Rv2159c_C81/84S) of Rv2159c were incubated in a buffer (100 mM KPi, pH 7.0, 1 mM EDTA, and 10 mM DTT) with and without DTT at 25°C for 0, 2, 4, and 6 min. The initial rates were corrected for the background oxidation of DTT in absence of the enzyme.

### Construction of sense and antisense Rv2159c

The sense and antisense oriented Rv2159c was cloned in pMV261 vector. The full length Rv2159c gene from *M. tuberculosis* was amplified using the respective oligonucleotide primers (Table S2). The amplified gene was inserted into pMV261 vector at 5′*BamH1* and 3′*EcoR1* sites to get Rv2159c in sense orientation (S-Rv2159c).

The antisense oriented Rv2159c clone was constructed in two steps. Firstly, the PCR amplified Rv2159c gene was cloned in pCR 2.1 TOPO vector with 5′*BamHI* and 3′*EcoRI* sites. Further, the Rv2159c gene insert was excised from pCR 2.1 TOPO vector with 5′*HindIII* and 3′*EcoRI* sites. Finally, the excised Rv2159c gene insert from pCR 2.1 TOPO vector was sub-cloned into pMV261 vector at 5′*EcoRI* and 3′*HindIII* sites to get Rv2159c in antisense orientation (As-Rv2159c). The constructed clones were checked for orientation with *SalI* restriction enzyme. Product size of ~780 bp confirms the clone in sense orientation and the product size of ~260 bp confirms the clone in antisense orientation. The S-Rv2159c and As-Rv2159c constructs were subjected to DNA sequencing to confirm their reading frames. The sequence confirmed clones as well as empty pMV261 vector (Rv) were then electroporated into electro competent *M. tuberculosis* H37Rv cells and plated on 7H10 agar media supplemented with OADC containing 20 μg/ml kanamycin.

### RNA isolation and real time qPCR

Total RNA was isolated from Rv, S-Rv2159c and As-Rv2159c strains at 0, 3, 5, 7, 14, and 21 days using an RNeasy kit (QIAGEN, Inc.,). The RNA was subsequently treated with DNaseI at 37°C for 45 min. The DNase was then inactivated by incubation at 75°C for 10 min. RNA was quantified by using a ND-1000 Nanodrop spectrophotometer (Nanodrop Technologies). Purified RNA was stored at −80°C. For determination of relative mRNA concentrations by qPCR, cDNA was synthesized with 1 μg of RNA using QuantiTect reverse transcription kit (Qiagen), according to the manufacturer's instructions. Quantitative RT-PCR primers for the Rv2159c and 16S rRNA genes were designed (Table S2). qPCRs were carried out using Taqman PCR master mix plus low ROX (Eurogentec) according to manufacturer's instructions using the Applied Biosystems 7300 real-time PCR system. To check for DNA contamination, control reactions for each sample were carried out in the absence of reverse transcriptase. The amplification conditions for all reactions were 1 cycle of 50°C for 2 min, 95°C for 10 min, followed by 40 cycles of 95°C for 15 s and 60°C for 1 min.

Analysis of qPCR data was carried out by relative quantification of Rv2159c gene expression using comparative CT method. For each qPCR run, the calculated threshold cycle (CT) was normalized to the CT of the internal control 16S rRNA gene amplified from the corresponding sample. Statistical analysis was carried out using GraphPad Prism software. The data presented are averages of three independent experiments and error bars represent standard deviations.

### Colony morphology

The Rv, S-Rv2159c and As-Rv2159c strains grown to mid-log phase in 7H9 medium were washed and serially diluted with phosphate-buffered-saline (PBS) containing 0.05% Tween 80. Samples of each 10-fold dilution were spotted onto middlebrook 7H10 and 7H11 agar plates containing 20 μg/ml kanamycin and incubated for 4 weeks at 37°C. Further, the plates were observed for difference in colony morphology.

### *In vitro* growth determinations

To determine the *in vitro* growth characteristics of Rv, S-Rv2159c and As-Rv2159c strains, log-phase cultures grown in 7H9-OADC-T medium with 20 μg/ml kanamycin were washed and diluted in fresh 7H9-OADC-T medium with appropriate antibiotics. The strains were incubated at 37°C with 150 rpm shaking. Aliquots of 100 μl were taken at 0, 3, 5, 7, 14, and 21 days for measuring OD at 600 nm using spectraMax 250 microplate reader (Molecular Devices). The viability of Rv, S-Rv2159c and As-Rv2159c strains were checked at each time point by plating the serial diluted cultures in 7H10-OADC agar plates and measuring colony forming units (CFUs).

### Estimation of viability under peroxide stress

The Rv, S-Rv2159c and As- Rv2159c strains were grown to mid-log phase in 7H9-OADC-T medium to an OD_600_ of 0.1 and exposed to peroxides such as CHP (Sigma) and hydrogen peroxide (Sigma) at a concentration of 0, 50, 100 μM and 0, 10, 20 mM respectively. Viability of the cultures were measured at 0, 4, and 24 h by measuring CFU.

### 2D gel electrophoresis

The Rv, S-Rv2159c and As-Rv2159c cell extracts were prepared and precipitated by SDS-trichloro acetic acid (TCA)–acetone method as reported earlier (Bisht et al., 2007; Sharma and Bisht, 2016). The pellets were air dried and suspended in appropriate volume of 2D-rehydration buffer (Bio-Rad, USA). The protein concentration was estimated using Bradford assay as reported earlier (Singhal et al., 2012).

Isoelectric focusing (IEF) was carried out using “in-gel rehydration” method. 7 cm immobilized pH gradient (IPG) strips of pH 4–7 (Bio-Rad, USA) were rehydrated overnight with 140 μg protein at 20°C. The strips were then focused on an IEF unit PROTEAN IEF Cell (Bio-Rad, USA) at 18°C using the following five-step program: (a) 0–250 V for 1.5 h in linear mode; (b) 250 V constant for 1.5 h in rapid mode; (c) 250–3000 V for 4 h in linear mode; (d) 3000 V constant until 15,000 Vh was reached and (e) 500 V constant at slow mode until the IPG strips were taken out from IEF cell. After IEF, strips were equilibrated at RT in equilibration buffer I and II (Bio-Rad, USA) for 5 min each and subjected to SDS-PAGE.

Gel images were acquired and analyzed by Chemidoc using Quantity One software (Bio-Rad, USA). Differentially expressed proteins were shortlisted by Student *t*-test using PDQuest software (Bio-Rad, USA). Protein spots that showed increased intensities with more than 1.5-fold were selected for identification.

### Mass spectrometry analysis

Differentially expressed protein spots were excised from the SDS-PAGE gels. In-gel digestion was performed for the protein spots as described (Shevchenko et al., 2006). The extracted peptides were purified using C-18 ZipTip pipette tips (Millipore, USA) and then spotted with 1:1 α-cyano-4-hydroxycinnamic acid matrix onto an Opti-TOF 96-well MALDI plates. Peptides were analyzed on a Matrix-assisted laser desorption ionization time-of-flight (MALDI-TOF MS/MS), Daltonics Ultraflex III mass spectrometer (Bruker). The resulting MS/MS spectra were analyzed using Mascot server (http://www.matrixscience.com).

### Statistical analysis

For *in vitro* growth kinetics and CFU studies, two-tailed ANOVA with multiple comparisons using Bonferroni post-test was used. For Alkylhydroperoxidase assay, one-tailed ANOVA with multiple comparisons using Bonferroni post-test was used.

## Results

### Cloning, expression and purification of Rv2159c

**Figure 1:**
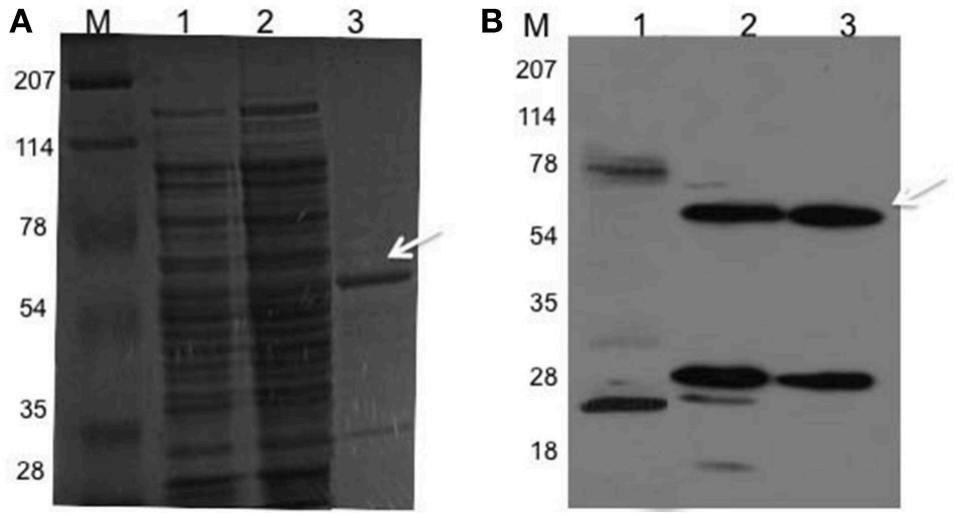
**Rv2159c cloning, expression and purification. (A,B)** SDS-PAGE (12% gel) separation of proteins and western immunoloblot with anti-GST antibody of *E. coli* BL21 cells expressed Rv2159c protein. Lane 1 shows the resolution of cytosolic proteins of BL21 cells (negative control); lane 2, BL21 cells expressed pDVA 2159-GST plasmid; lane 3 shows the purified recombinant GST-Rv2159c protein using glutathione sepharose affinity chromatography and lane M, protein molecular weight ladder.

The 1035 bp coding region of Rv2159c from *M. tuberculosis* was inserted at *BamH1* and *EcoR1* sites of pGEX 4T-1 vector. Restriction digestion and DNA sequencing confirmed plasmid was transformed into *E. coli* BL21 (DE3) cells for expression of Rv2159c as GST fusion protein. The construct expressed a high proportion of Rv2159c protein after 4–5 h of induction. The recombinant Rv2159c protein was purified to near homogeneity using glutathione sepharose affinity chromatography. Molecular mass of the purified recombinant protein was showed at 60 kDa, which corresponds the expected molecular mass of Rv2159c protein (Figures 1A,B). For some unknown reason, the free GST tag was also co-purified with Rv2159c protein as 28 kDa protein band (Figure 1B). One possible reason could be the GST fusion tag might have degraded upon denaturation and reduction during protein gel electrophoresis.

### Interaction between PknI and Rv2159c mutants

**Figure 2:**
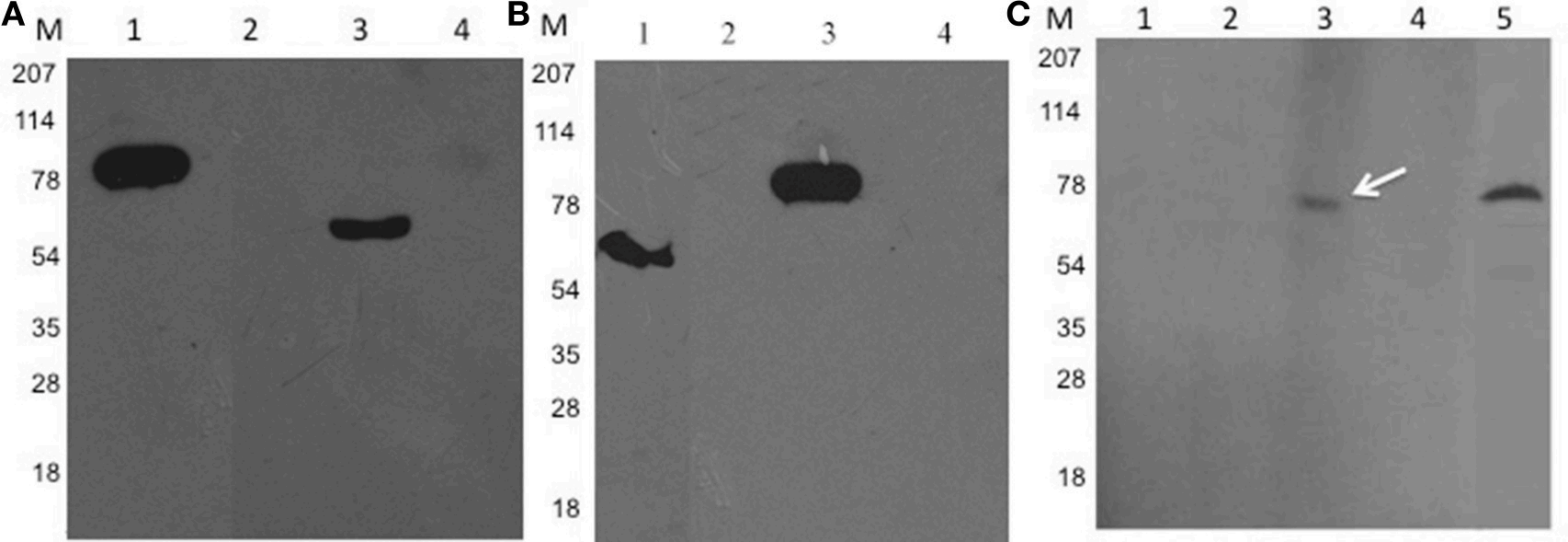
**PknI interacts with Rv2159c. (A,B)** Pull down assay confirms the interaction between PknI and Rv2159c. Cytoplasmic crude lysates containing recombinant His-PknI and GST-Rv2159c proteins were incubated with Ni-NTA resin (**A**, lane 1) or glutathione sepharose resin respectively (**B**, lane 1). Unbound proteins (lane 2) were washed off and incubated with their respective substrates. The protein complexes were eluted (lane 3) and immunoblotted with anti-GST **(A)** and anti-His antibodies **(B)** respectively. As a control, the bait proteins were incubated with bovine serum albumin (BSA) protein (lane 4). Lane 1 from **(A)** and **(B)** shows the bait protein load controls of PknI and Rv2159c proteins that are probed against anti-His **(A)** and anti-GST **(B)** antibodies respectively. Several lanes were merged for photogenic purpose. **(C)** FWB identifies the crucial amino acids in PknI-Rv2159c interaction. The purified GST-Rv2159c point mutants A49P (lane 1), G50A (lane 2), W51A (lane 3) and 49AGW51 (lane 4) along with purified Rv2159c protein (lane 5) were incubated with His-PknI protein and subsequently probed with anti-His antibody. Lane M, Molecular mass standard.

In our previous study, we have shown the protein kinase PknI interacts with Ala-Gly-Trp motif residues of Rv2159c (Venkatesan et al., 2015). We expanded our previous observations and confirmed the results with pull down assay using recombinant His-PknI and GST-Rv2159c proteins. Ni-NTA affinity chromatography with recombinant His-PknI protein was able to pull down GST-Rv2159c protein (Figure 2A, lane 3). In a reverse reaction, glutathione sepharose affinity chromatography with recombinant GST-Rv2159c protein was also observed to pull down His-PknI protein (Figure 2B, lane 3). In a control assay, the recombinant proteins did not pull down the tag free BSA (Figure 2, lane 4). Surprisingly, no protein phosphorylation was observed between PknI and Rv2159c (Data not shown).

To identify the residues of Rv2159c contributing to the protein interaction, the possible binding residues (Ala49, Gly50, Trp51) were mutated to proline and alanine amino acids respectively. Further, the GST-Rv2159c variants (A49P, G50A, W51A, 49AGW51) were subjected to FWB and probed with recombinant His-PknI protein. Mutation of A49P and G50A amino acids of Rv2159c protein totally abolished the interaction with PknI protein. However, W51A amino acid mutation did not affect its interaction with PknI (Figure 2C). These interacting residue mutants were inactive for phosphorylation by PknI as WT Rv2159c (Data not shown). Together, these results confirm that only Ala49 and Gly50 amino acids of Rv2159c protein contribute to its interaction with PknI and no phosphorylation was involved in the interaction.

### Catalytic activity of Rv2159c

To identify the importance of two cysteine residues in catalytic site of Rv2159c protein (C-X-X-C), the cysteine amino acids were mutated to serine using site directed mutagenesis. The constructed point mutants Rv2159c_C81S, Rv2159c_C84S, Rv2159c_C81/84S were verified with DNA sequencing. The confirmed plasmids were transformed into *E. coli* BL21 (DE3) cells and the mutant proteins were purified to homogeneity.

**Figure 3:**
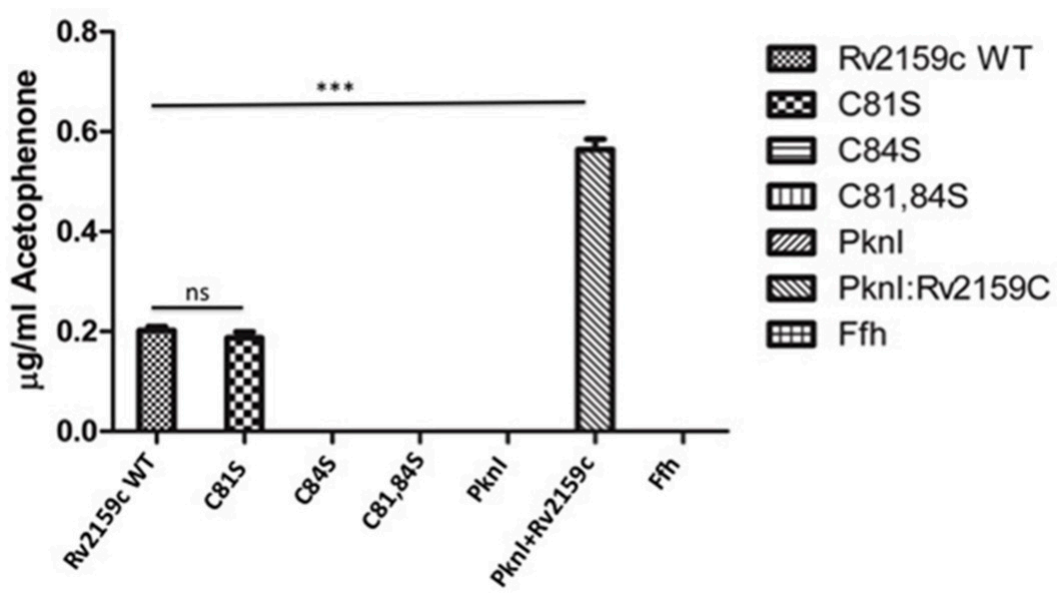
**Catalytic activity of Rv2159c and its mutants**. Alkyl hydroperoxidase activity of Rv2159c variants (WT, C81S, C84S and C81,84S) were detected by measuring acetophenone using HPLC. The peroxidase activity of Rv2159c was also checked in the presence and absence of its substrate, PknI. No catalytic activity was observed with non-specific protein (Ffh), PknI alone and buffer control. One way ANOVA was performed. ^***^ Indicates *P* < 0.001.

The catalytic activity of Rv2159c WT and its catalytic site mutants (Rv2159c_C81S, Rv2159c_C84S, and Rv2159c_C81/84S) was detected by incubating the purified proteins with the substrate, CHP and the decomposed product acetophenone was measured using HPLC. Measurement of Rv2159c dependent alkylhydroperoxidase activity toward CHP revealed that the activity of Rv2159c_C81S mutant was similar to that of WT, whereas no activity was detected with Rv2159c_C84S mutant. Surprisingly, the interaction of Rv2159c WT with PknI resulted in 3-fold increase of Rv2159c's peroxidase activity (Figure 3). These results demonstrate that Rv2159c_C84S residue is responsible for Rv2159c's catalytic activity.

We next examined the possibility of two cysteine amino acids to form disulfide bridges during the catalytic activity of Rv2159c. The WT and catalytic site mutants were treated with redox agent DTT in the presence of *tert*-butyl hydroperoxide and the oxidation of cysteines were measured. No cysteine oxidation was detected with the Rv2159c variants suggesting the protein does not form any intramolecular disulfide bridges (Figure S1).

### Antisense inhibition of Rv2159c

**Figure 4:**
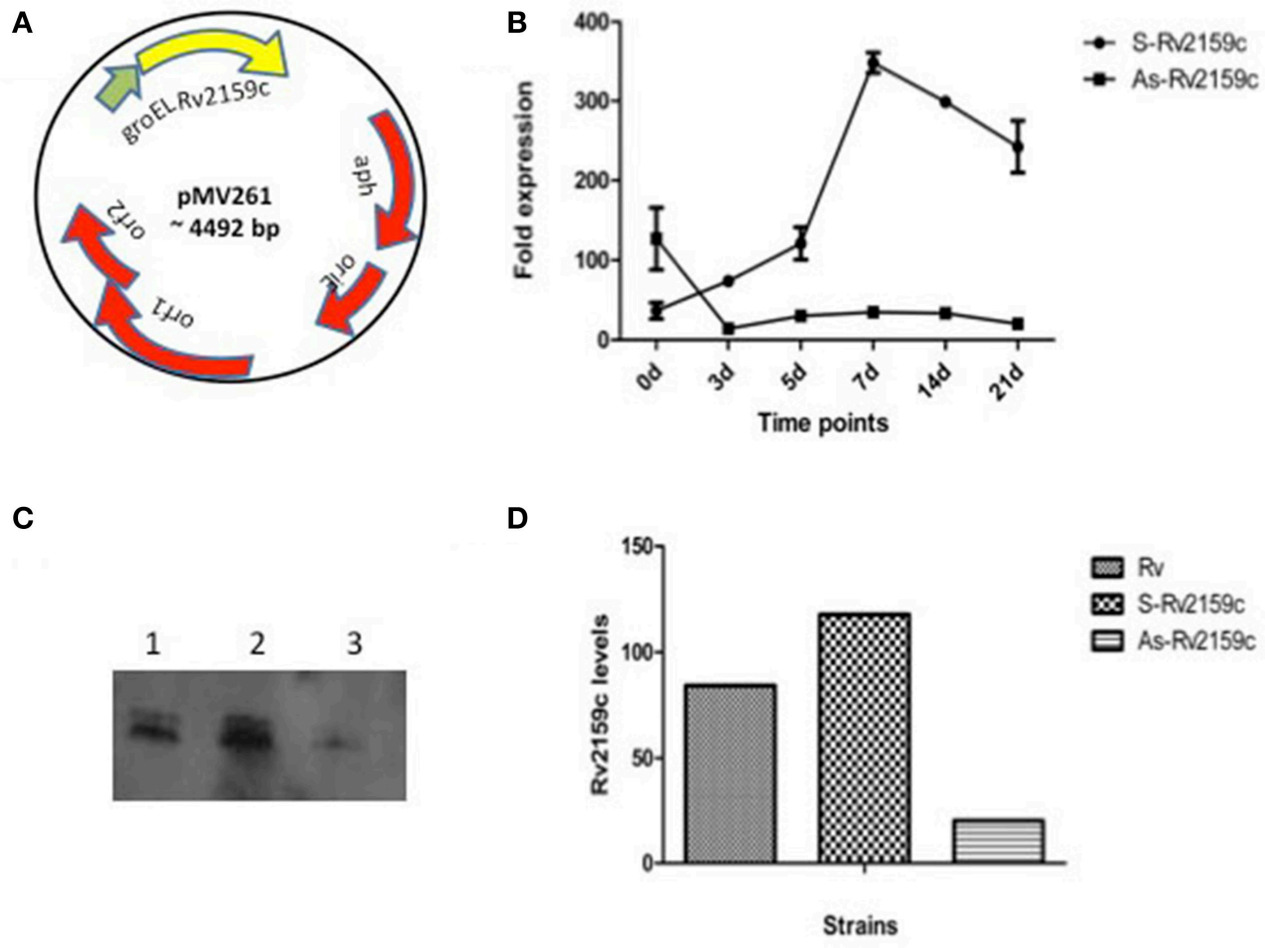
**Differential expression of Rv2159c. (A)** pMV261 plasmid harboring Rv2159c in sense (S-Rv2159c) and antisense (As-Rv2159c) orientation. **(B)** Quantification of Rv2159c expression: Validation of Rv2159c differentially expressing constructs using quantitative real time PCR. Cell lysate of *M. tuberculosis* over-expressing (S-Rv2159c) and under-expressing (As-Rv2159c) Rv2159c gene was quantified at 0, 3, 5, 7, 14, and 21 days against the house keeping gene 16S rRNA. **(C)** FWB analysis for Rv2159c differentially expressing strains. Equal amount (50 μg) of cytoplsmic lysates from 21 d cultures of Rv (lane 1), S-Rv2159c (lane 2) and As-Rv2159c (lane 3) strains were probed with bait protein, PknI and anti-His antibody. **(D)** Quantification of Rv2159c protein band from Rv, S-Rv2159c and As-Rv2159c strains using densitometer scanning.

In order to understand the physiological importance of Rv2159c, the differentially expressing Rv2159c strains were generated in *M. tuberculosis* strain. For this, the sense and antisense DNA strands were cloned in pMV261 vector under the *hsp* promoter (Figure 4A). Quantification of Rv2159c transcript in S-Rv2159c and As-Rv2159c strains at different time points (0, 3, 5, 7, 14, and 21 d) showed that the S-Rv2159c was induced to 347-fold on 7 d and later depleted to 242-fold on 21 d. While the As-Rv2159c strain depleted to 14-folds on 3 d and maintained to 20-fold on 21 d (Figure 4B). The differentially expressing strains were also quantified at protein level using FWB. When equal concentration of protein preparations from Rv, S-Rv2159c and As-Rv2159c strains were probed with PknI protein, a 33.04% increase in S-Rv2159c and 122.17% decrease in As-Rv2159c strains were observed as compared to Rv (Figures 4C,D). Together, these results validated the differentially expressing Rv2159c strains.

### *In vitro* growth of the Rv2159c strains

**Figure 5:**
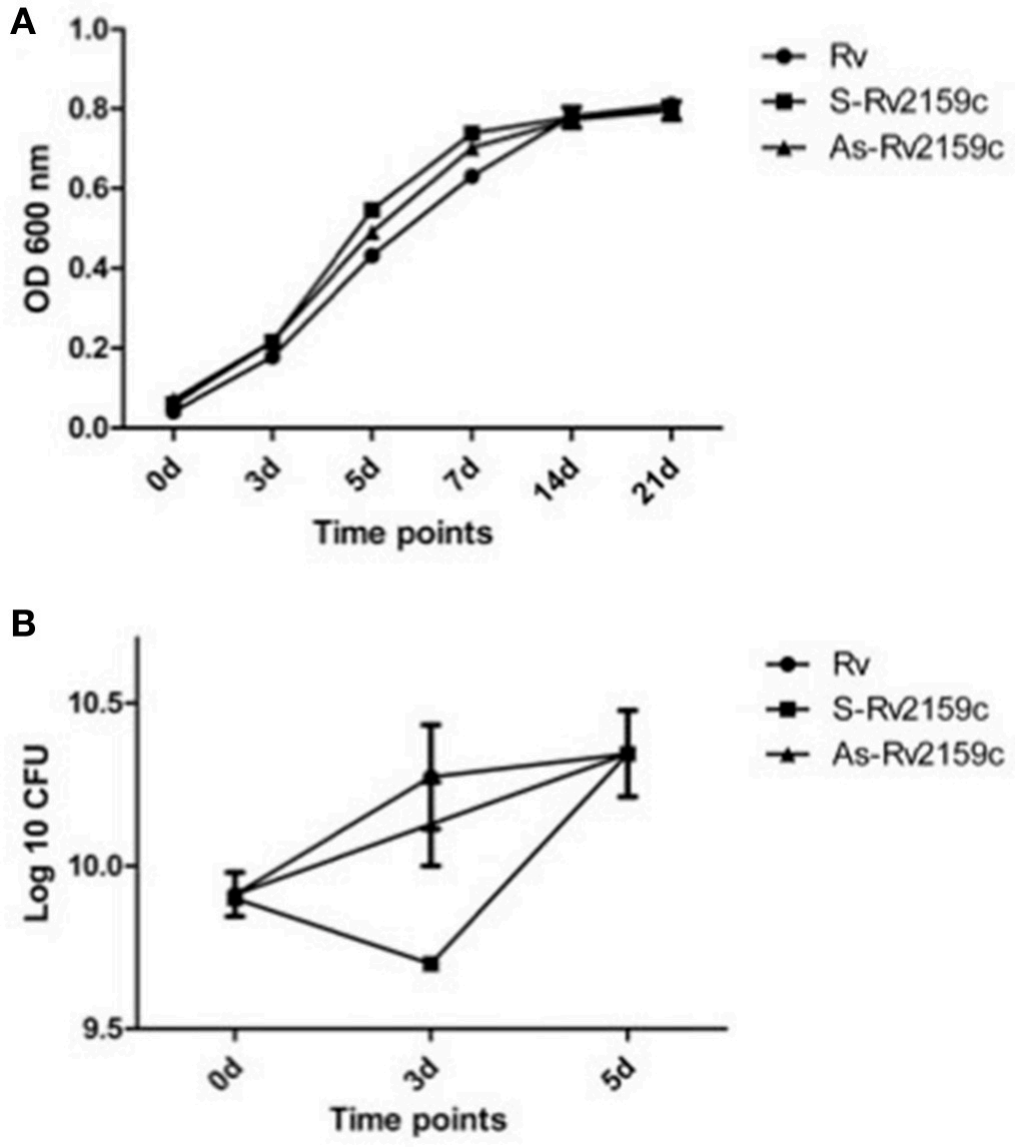
*****In vitro*** growth kinetics of Rv2159c. (A)** The S-Rv2159c and As-Rv2159c constructs in *M. tuberculosis* were grown in 7H9 supplemented medium and the growth kinetics were monitored at 0, 3, 5, 7, 14, and 21 d. Graph represents the OD_600_ at different time points and represent by their mean ± SEM from three different experiments. Comparison of Rv vs. S-Rv2159c showed a significance of *P* < 0.001 at 5 and 7 d, comparison of Rv vs. As-Rv2159c showed a significance of *P* < 0.05 at day 5 and *P* < 0.01 at day 7. The statistical significance calculated by two way ANOVA using graph pad prism. **(B)** Survival kinetics of Rv2159c differentially expressing strains in *M.tuberculosis*. The S-Rv2159c and As-Rv2159c constructs in *M. tuberculosis* were plated in complete 7H10 agar plate and CFUs were measured at 0, 3, and 5 day and represent by their mean ± SEM from three different experiments. Comparison of Rv vs. S-Rv2159c showed a significance of *P* < 0.05 at day 3.

To determine whether the differential expression of Rv2159c has brought any changes in the *in vitro* growth, we compared the growth profiles of Rv, S-Rv2159c and As-Rv2159c strains by monitoring OD_600_ and quantification of CFUs at 0, 3, 5, 7, 14, and 21 d. No altered growth rates were observed between the strains during OD_600_ and survival CFU (Figures 5A,B). Analysis of the colony morphology for differentially expressing Rv2159c strains resulted in no differential colony pattern in both middlebrook 7H10 and 7H11 medium (Data not shown).

### Role of Rv2159c in protection against peroxides

**Figure 6:**
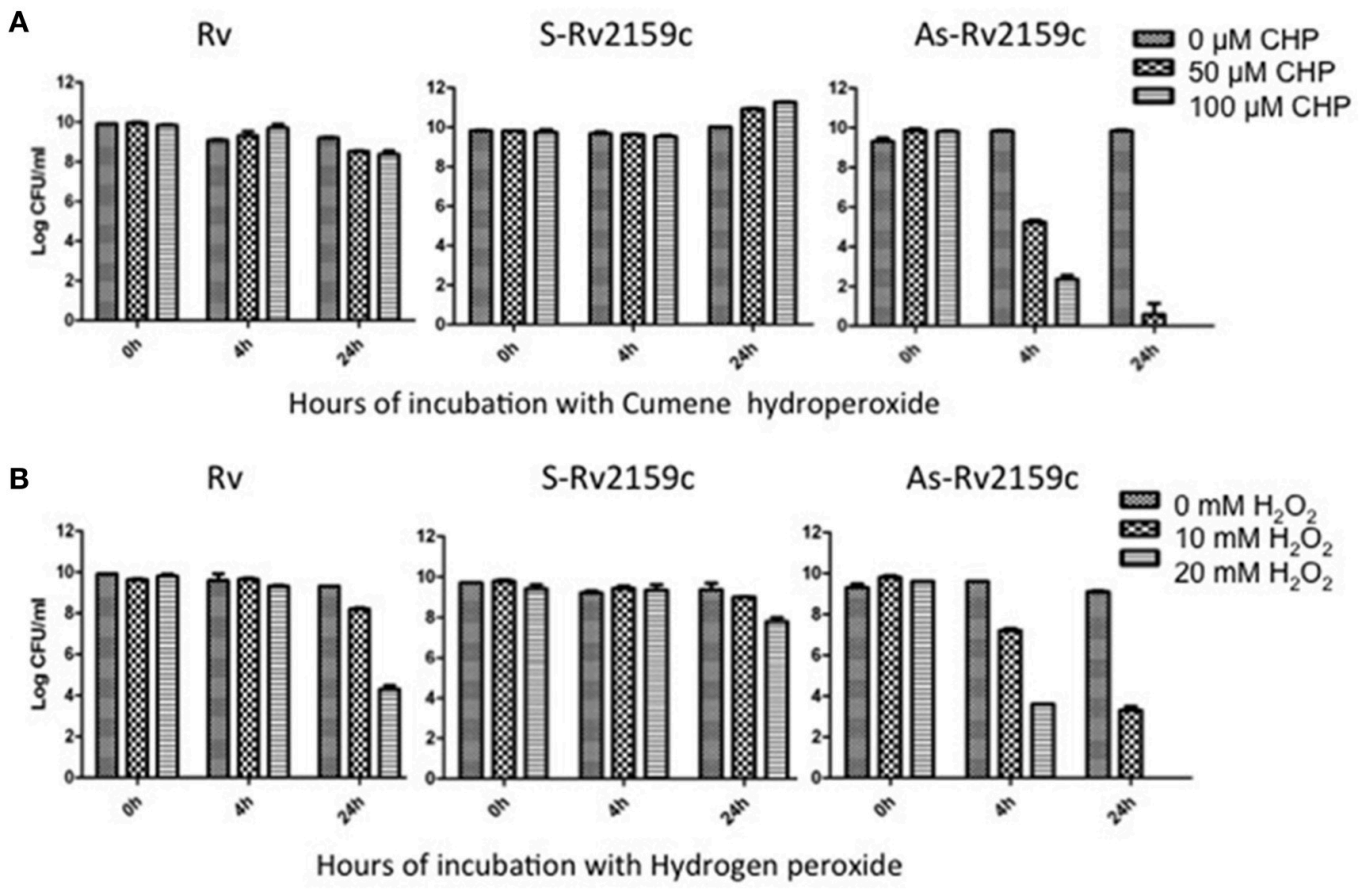
**Effect of peroxides on S-Rv2159c and As-Rv2159c. (A)** The Rv, S-Rv2159c and As-Rv2159c constructs in *M. tuberculosis* were diluted to an OD_600_ of 0.1 in complete 7H9 medium and CFU was measured at 0, 4, and 24 h post exposure with **(A)** 0, 50 and 100 μM of CHP and **(B)** 0, 10 and 20 mM hydrogen peroxide.

To understand the role of Rv2159c in protecting *M. tuberculosis* against oxidative stress, the Rv, S-Rv2159c and As-Rv2159c strains were exposed to CHP and hydrogen peroxide and CFU were determined at 0 h, 4 h and 24 h as a measure of cell viability. Exposure of S-Rv2159c to 50 and 100 μM of CHP showed increased growth rate at 24 h. However, exposure of As-Rv2159c to CHP showed significant decrease in growth at 4 h and complete sterilization of the culture at 24 h in response to 100 μM CHP (Figure 6A). Exposure of S-Rv2159c to 10 mM hydrogen peroxide showed no difference in growth rate as compared to Rv. Whereas, exposure of As-Rv2159c to hydrogen peroxide showed decreased growth rate and complete sterilization to 20 mM hydrogen peroxide (Figure 6B). These results suggest that the Rv2159c gene product plays important role in oxidative stress survival of *M. tuberculosis*.

### Rv2159c substrate identification

**Figure 7:**
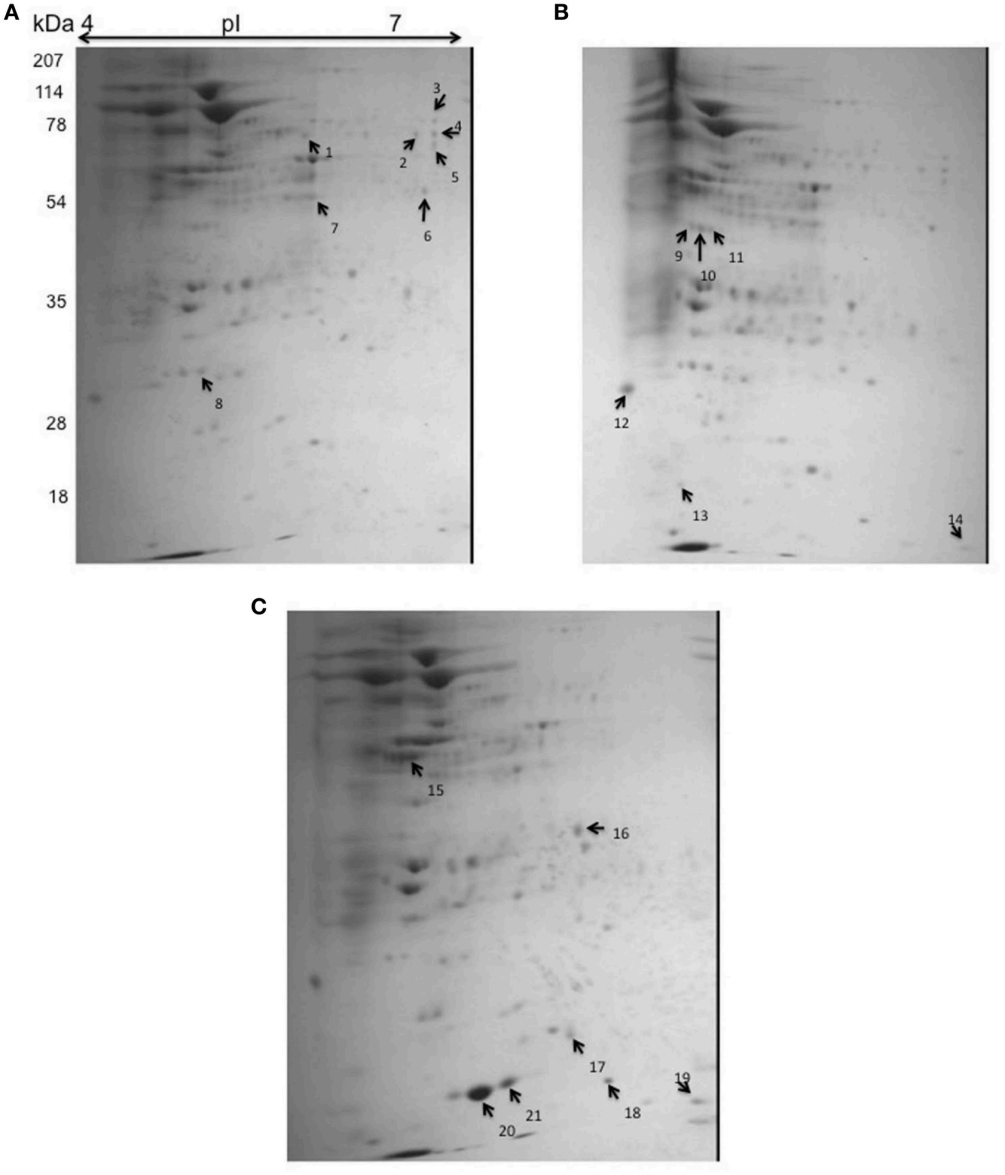
**2D gel electrophoresis for S-Rv2159c and As-Rv2159c strains**. The cytoplasmic cell extracts of S-Rv2159c, As-Rv2159c and Rv constructs in *M. tuberculosis* were subjected to 2D gel electrophoresis. The CBB R250 stained gels **(A)** Rv, **(B)** S-Rv2159c, and **(C)** As-Rv2159c were shown. The arrow marks represents the difference in spots using PDQuest software were analyzed by mass spectrometry.

In order to identify the putative target genes of Rv2159c, the cytoplasmic lysates of Rv, S-Rv2159c and As-Rv2159c strains were separated by 2-D gel electrophoresis. Statistical analysis of the gels detected 21 spots with significant changes between Rv versus S-Rv2159c and Rv versus As-Rv2159c (Figure 7). A total of 12 protein spots could be identified using MALDI TOF TOF MS/MS analysis and they were categorized into five functional categories based on the information from Tuberculist and UniProt databases (Table 1). Most of the identified proteins were involved in intermediary metabolism and stress regulations. The other functional groups such as cell wall processes and lipid metabolism related proteins are also differentially expressed.

**Table 1:** **Identification of the differentially regulated 2D protein spots of Rv, S-Rv2159c, and As-Rv2159c**.

**Spot no**.	**Gene name**	**Accession no**.	**Mascot score**	**No. of matched peptides**	**Gene index**	**Theoretical pI/kDa**	**Expression**
**GROUP 1: INTERMEDIARY METABOLISM, RESPIRATION**							
4	Uncharacterised oxidoreductase, GuaB1	Rv1843c	69	1	gi 492067680	6.29/49.93	Absent in AsRv2159c
8	ATP-dependent CLP protease protelytic subunit 2, ClpP2	Rv2460c	74	4	gi 15609597	4.75/23.5	Absent in As-Rv2159c
9, 10, 11	Malate dehydrogenase, Mdh	Rv1240	496	4	gi 489501402	4.39/34.28	More in S-Rv2159c
12	Glycogen accumulation regulator, GarA	Rv1827	457	3	gi 240104237	4.06/17.21	More in S-Rv2159c
**GROUP 2: VIRULENCE, DETOXIFICATION AND ADAPTATION**							
16	Universal stress protein family protein, TB31.7	Rv2623	800	6	gi 685981944	5.55/31.65	Present in AsRv2159c
18	Iron-regulated universal stress protein family protein, TB15.3	Rv1636	243	3	gi 669108828	5.62/15.31	More in AsRv2159c
20, 21	Heat shock protein, HspX	Rv2031c	327	3	gi 801196443	4.75/16.22	Present only in AsRv2159c
**GROUP 3: LIPID METABOLISM**							
7	Fatty acid CoA synthetase, FaD17	Rv3506	79	2	gi 764899599	4.93/53.73	Less in As-Rv2159c
14	3-hydroxyacyl-ACP dehydratase subunit, HadB	Rv0636	83	1	gi 686051919	6.52/14.93	More in S-Rv2159c
**GROUP 4: CELL WALL AND CELL PROCESSES**							
15	Conserved protein with FHA domain, FhaA	Rv0020c	399	3	gi 15607162	4.65/56.88	More in AsRv2159c
**GROUP 5: INFORMATION PATHWAY**							
6	Ribonuclease HII protein, RnhB	Rv2902c	64	3	gi 686045826	10.47/27.64	Absent in AsRv2159c

In comparison to Rv, over-expression of Rv2159c (S-Rv2159c) leads to high abundance of two metabolic pathway proteins such as, Rv1240 (Malate dehydrogenase Mdh), Rv1827 (Glycogen accumulation regulator GarA) and low abundance of fatty acid synthesis (FAS) intermediate, Rv0636 (3-hydroxyacyl-ACP dehydratase subunit HadB). Interestingly, down regulation of Rv2159c (As-Rv2159c) leads to the total absence of Rv1843c (GuaB1), an Oxidoreductase. The other proteins that are down regulated are Rv2460c (ATP-dependent CLP protease protelytic subunit 2 ClpP2), Rv3506 (Fatty acid CoA synthetase FaD17) and Rv2902c (Ribonuclease HII protein RnhB). The three stress regulated proteins, Rv2031c (Heat shock protein HspX), Rv2623 (Universal stress protein family protein TB31.7), Rv1636 (Iron-regulated universal stress protein TB15.3) and a cell wall associated protein, Rv0020c (Conserved protein with Fork Head Associated (FHA) domain FhaA) were noticed to be abundant in antisense Rv2159c strain. In summary, these analyses suggest that Rv2159c plays an important role in regulating intermediate metabolic pathways.

## Discussion

In this study, we report for the first time purification, interaction with PknI and catalytic activity of the heterologously expressed Rv2159c protein. The critical residues involved in interaction between Rv2159c and PknI was identified and confirmed. We were able to understand the role of Rv2159c of *M. tuberculosis* using antisense expression studies and we have achieved its phenotypic characterization and substrate identification.

Rv2159c was over-expressed heterologously in soluble form as a N-terminal GST fusion protein. Mutation of two catalytic site cysteine amino acid residues (Cys81 and Cys84) identified the importance of Cys84 in alkylhydroperoxidase activity. Interestingly, the peroxidase activity of Rv2159c protein was increased to 3-fold upon its interaction with PknI (Figure 3). The PknI-Rv2159c interaction is mediated through phosphorylation independent physical interaction between two proteins. Mutation of active site residues of Rv2159c identified that Ala49 and Gly50 amino acids were responsible for the interaction (Figure 2C). In addition, like WT Rv2159c, no phosphorylation was observed with active site mutants of Rv2159c. These mutations could have changed the structural fold of the Rv2159c protein, so that the A49P and G50A Rv2159c mutants could not interact with PknI protein. However, no difference in interaction pattern was observed with catalytic site mutants (Data not shown). This denotes that the PknI interacts through the active site (Ala-Gly) of Rv2159c protein and increases its catalytic activity. To our surprise, though the Rv2159c protein is a peroxidase, it did not form disulfide bridges between the two catalytic site cysteines. Whereas, the two other alkylhydroperoxidase proteins of *M. tuberculosis*, AhpC and AhpD were shown to form disulfide bridges (Hillas et al., 2000).

Though the gene, Rv2159c is non-essential in *M. tuberculosis*, we have employed antisense strategy to elucidate its role in *M. tuberculosis*. However, construction of gene knockout for Rv2159c is under investigation. The use of pMV261 vector in antisense gene knockdown of *M. tuberculosis* genes has been elucidated previously (Wu et al., 2004; Sun et al., 2010; Kunisch et al., 2012). Cloning the coding sequence of sense and antisense oriented Rv2159c under *hsp60* promoter of *E. coli*-mycobacteria shuttle vector controlled the expression of Rv2159c. qRT-PCR and FWB assessed the expression of S-Rv2159c and As-Rv2159c strains. qRT-PCR quantification of S-Rv2159c strain induced Rv2159c from day 3 and reached its maximum expression on day 7 with 347-fold expression and the As-Rv2159c down regulated from day 3 with 14-fold expression. The differentially expressing strains from 21 d culture were also quantified at protein level by probing with its interacting partner, PknI. Densitometer measurements validated the S-Rv2159c and As-Rv2159c constructs with 33.04% increase and 122.17% decrease in expression respectively (Figure 4). However, no difference in Rv2159c expression was noticed when the strains were exposed with and without heat induction at 42°C to induce the *hsp* promoter (Data not shown).

There were no morphological changes associated with colony morphology of differentially expressing Rv2159c strains. The differential expressing Rv2159c strains did not differ in their growth kinetics. Interestingly, knockdown of Rv2159c showed decrease growth rate, which denotes its sensitivity against CHP and hydrogen peroxide stresses. In the other hand, over-expression of Rv2159c resulted in increased growth rate of the bacilli (Figure 6). Thus, the differential growth rate of Rv2159c strains suggested that the gene is responsible for protection against oxidative stresses of the bacteria.

Out of 21 differentially expressed proteins identified by 2-D gel electrophoresis, only 12 proteins could be identified using MALDI TOF-TOF MS/MS analysis. The remaining 9 proteins spots could not be identified because the amount of proteins was too small and no satisfactory spectrum was available. Over-expression of Rv2159c induces metabolic pathway related proteins, suggesting a possible role of Rv2159c in *M. tuberculosis* metabolism. Both the substrates, Mdh (Rv1240) and GarA (Rv1827) are TCA cycle regulators, where the malate dehydrogenase oxidizes the conversion of malate to oxaloacetate and glycogen accumulation regulatory protein GarA (Rv1827), acts as a key metabolic intermediate for the enzymes that use α-ketoglutarate (Ventura et al., 2013; Wang et al., 2015). Interestingly, over-expression of Rv2159c accumulated type II FAS intermediate HadB (Rv0636) and under-expression of Rv2159c depleted type I FAS intermediate, FaD17 (Rv3506).

Loss of Rv2159c induces three stress proteins, in which the 16 kDa heat shock protein HspX (Rv2031c) and universal stress protein family protein TB 31.7 aids in preventing misfolding of *M. tuberculosis* proteins under stress conditions. Both the HspX (Rv2031c) and TB 31.7 (Rv2623) also showed increase in their expression during dormancy conditions (Drumm et al., 2009; Dubaniewicz et al., 2013). However, the iron-regulated universal stress protein, TB15.3 (Rv1636) was shown to provide only a generalized stress response. Another protein that was down regulated in As-Rv2159c strain was also shown to be important in bacterial stresses. The proteolytic degradation component, ClpP2 (Rv2460c) is a core subunit of proteasome complex and it was observed to be up regulated under stress conditions (Mehra and Kaushal, 2009). Down regulation of Rv2159c also induced a FHA domain containing protein Rv0020c. FhaA is a substrate of cell division regulator protein, PknB (Roumestand et al., 2011).

The absence of GuaB1 (Rv1843c) was unique to the proteomic profile of As-Rv2159c. The two orthologs of GuaB1 such as GuaB2 and GuaB3 together forms an operon, whereas the GuaB1 is present elsewhere in the genome. Biochemical characterisation of GuaB ortholog suggested the potential role of GuaB2 in Inosine monophosphate dehydrogenase activity, whereas the role of GuaB1 and GuaB3 is still unknown (Usha et al., 2011). The gene ontology of molecular function classifies GuaB1 as an uncharacterized oxidoreductase. Taken together, we hypothesize that the oxidoreductase, GuaB1 (Rv1843c) could be a probable substrate for the alkylhydroperoxidase, Rv2159c and they both could act together during oxidative stresses. But, identification of Rv2159c substrates under oxidative stress conditions would completely unravel the other probable substrates of Rv2159c.

Protein kinases control every aspect of cellular functions through signal transduction. The STPK, PknI was shown to be involved in cell division and virulence of *M. tuberculosis* (Kandasamy et al., 2014). Deletion of the *pknI* gene leads to increased bacillary load in severe combined immunodeficiency (SCID) mice organs than its parental strain. In addition, the *pknI* deletion mutant was reported to grow well in low pH and low oxygen conditions that mimics the macrophage environment (Gopalaswamy et al., 2009). This study addresses the role of PknI in regulating *M. tuberculosis* during oxidative stress condition. Our results indicates that PknI as a kinase protein senses the hypoxic environment of the bacteria and interacts with redox protein Rv2159c in cytosol and thereby maintains the cellular homeostasis.

In summary, Rv2159c adds to the peroxidase group of proteins in *M. tuberculosis*. Protein interaction and phosphorylation studies showed that the Rv2159c protein communicates with PknI only through Ala49 and Gly50 amino acids and there is no phosphorylation involved in the interaction. Also, interaction with PknI rapidly increased Rv2159c's peroxidase activity. Knockdown of Rv2159c leads to total susceptibility to peroxides. Our studies also indicated that, Rv2159c regulates various functions such as intermediate metabolism, lipid metabolism and stress regulations await further confirmation. Experiments are in progress to determine the virulence of Rv2159c in *in vitro* and *in vivo* systems.

## Author contributions

SN: Conceived and supervised the study; AV and KP: Designed experiments; AV: Conducted most of the experiments, analyzed the results and wrote the manuscript. DS: Conducted the experiments involving 2D gel electrophoresis; DB: Provided technical support for performing 2DIGE.

### Conflict of interest statement

The authors declare that the research was conducted in the absence of any commercial or financial relationships that could be construed as a potential conflict of interest.

## Supplementary material

Figure S1 **DTT oxidation assay for Rv2159c**. Quantification of cysteine oxidation with Rv2159c WT and its catalytic site mutants (C81S, C84S, C81/84S) in the presence of DTT.

Table S1 **List of constructs used in this study**.

Table S2 **Details of primers used in this study**.
