# Supplementary figures and images for: Functional Characterization of PknI-Rv2159c Interaction in Redox Homeostasis of Mycobacterium tuberculosis
Source: Front Microbiol. 2016 Oct 21;7:1654. doi: 10.3389/fmicb.2016.01654 (PMC5073100; doi:10.3389/fmicb.2016.01654)

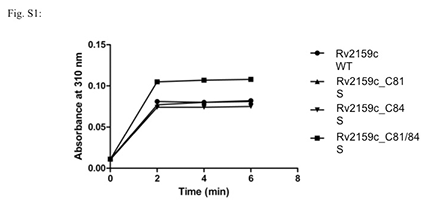

Supplement: Figure S1 — DTT oxidation assay for Rv2159c. Quantification of cysteine oxidation with Rv2159c WT and its catalytic site mutants (C81S, C84S, C81/84S) in the presence of DTT. [file Image1.TIF]
