# Supplementary material for: Functional Characterization of PknI-Rv2159c Interaction in Redox Homeostasis of Mycobacterium tuberculosis
Source: Front Microbiol. 2016 Oct 21;7:1654. doi: 10.3389/fmicb.2016.01654 (PMC5073100; doi:10.3389/fmicb.2016.01654)
Supplement: Table S1 — List of constructs used in this study. [file Table1.DOCX]

**Table S1:**

List of constructs used in this study:

| Strain or plasmid | Source or description | Reference or source |
| --- | --- | --- |
| *E. coli* DH5α | F^-^ *end*A1 *gln*V44 *thi-1 rec*A1 *rel*A1 *gyr*A96 *deo*R *nup*G ϕ80d*lacZΔ*M15 Δ(*lacZYA-argF*)U169, *hsd*R17(rK- mK+), λ- | Invitrogen |
| *E. coli* BL21 (DE3) | F^-^ *ompT gal dcm lon hsdS_B_ (r_B_^-^ m_B_^-^*) λ(DE3 [*lac*I *lac*UV5-T7 *gene* 1 *ind*1 *sam7 nin5*]) | Invitrogen |
| *M. tuberculosis* H37Rv | Lab stock |  |
| Plasmids |  |  |
| pGEX 4T-1 | lacT^q^, 4.9 kb, Amp^r^, GST gene fusion vector | GE healthcare |
| pCR 2.1 | TOPO cloning vector, Kan^r^, Amp^r^ | Invitrogen |
| pMV261 | *E. coli* mycobacterial shuttle vector, Kan^r^, *hsp60* promoter | [1] |
| pDVA 2159-GST | pGEX 4T-1 vector carrying the coding region of Rv2159c gene from *M. tuberculosis*, Amp^r^ | This study |
| pDVA 2159-S | pMV 261 vector carrying the coding region of Rv2159c gene from *M. tuberculosis* in sense orientation, Kan^r^ | This study |
| pDVA 2159-S TOPO | pCR 2.1 vector carrying the coding region of Rv2159c gene from *M. tuberculosis* in sense orientation, Amp^r^ | This study |
| pDVA 2159-As | pMV 261 vector carrying the coding region of Rv2159c gene from *M. tuberculosis* in antisense orientation, Kan^r^ | This study |
| pDVA 2159-C81S | pGEX 4T-1 vector carrying the C81S mutation in Rv2159c gene from *M. tuberculosis*, Amp^r^ | This study |
| pDVA 2159-C84S | pGEX 4T-1 vector carrying the C84S mutation in Rv2159c gene from *M. tuberculosis*, Amp^r^ | This study |
| pDVA 2159-C81/84S | pGEX 4T-1 vector carrying the double mutant at C81/84S in Rv2159c gene from *M. tuberculosis*, Amp^r^ | This study |
| pDVA 2159-A49P | pGEX 4T-1 vector carrying the A49P mutation in Rv2159c gene from *M. tuberculosis*, Amp^r^ | This study |
| pDVA 2159-G50A | pGEX 4T-1 vector carrying the G50A mutation in Rv2159c gene from *M. tuberculosis*, Amp^r^ | This study |
| pDVA 2159-W51A | pGEX 4T-1 vector carrying the W51A mutation in Rv2159c gene from *M. tuberculosis*, Amp^r^ | This study |
| pDVA 2159-AGW | pGEX 4T-1 vector carrying the AGW mutation in Rv2159c gene from *M. tuberculosis*, Amp^r^ | This study |

**References:**

[1] Stover, C.K., de la Cruz, V.F., Fuerst, T.R., Burlein, J.E., Benson, L.A., Bennett, L.T., et al. New use of BCG for recombinant vaccines. Nature. 1991, 351, 456-60.
