# Supplementary material for: Functional Characterization of PknI-Rv2159c Interaction in Redox Homeostasis of Mycobacterium tuberculosis
Source: Front Microbiol. 2016 Oct 21;7:1654. doi: 10.3389/fmicb.2016.01654 (PMC5073100; doi:10.3389/fmicb.2016.01654)
Supplement: Table S2 — Details of primers used in this study. [file Table2.DOCX]

**Table S2:**

Details of primers used in this study:

| Primer name | Sequence (5'-3') | Purpose |
| --- | --- | --- |
| 2159 FP | CCC**GGATCC**CCAGGAGGGAGTCGAATCATGAA | Cloning |
| 2159 RP | CCC**GAATTC**GGCCAGCGATGACACCCTACC | Cloning |
| 2159 RT FP | CACACACCACCATGCTGTAC | qRT-PCR |
| 2159 RT RP | GCGTGCGATGAAGTGGAATT | qRT-PCR |
| 2159 Probe | FAM-GATGTCGCCGCCGAATAC-TAMRA | qRT-PCR |
| 16S rRNA FP | TGAGATGTTGGGTTAAGTCCCGCA | qRT-PCR |
| 16S rRNA RP | TAGCATGTGTGAAGCCCTGGACAT | qRT-PCR |
| 16S rRNA probe | FAM-ACGAGCGCAACCCTTGTCTCATGTT-TAMRA | qRT-PCR |
| 2159-C81S FP | GCCAGCCTGCGC**TCC**CCCTGGTG | SDM |
| 2159- C81S RP | CACCAGGGGGAGCGCAGGCTGGC | SDM |
| 2159- C84S FP | GCTGCCCCTGG**TCC**GTCGACGCA | SDM |
| 2159- C84S RP | TGCGTCGACGGACCAGGGGCAGC | SDM |
| 2159-C81/84S FP | GCCTGCGC**TCC**CCCTGG**TCC**GTCGAC | SDM |
| 2159- C81/84S RP | GTCGACGGACCAGGGGGAGCGCAGGC | SDM |
| 2159-A49P FP | cgcccagccgggggtgagcagtc | SDM |
| 2159-A49P RP | gactgctca**ccc**ccggctgggcg | SDM |
| 2159-G50A FP | acgtcgcccaggcggcggtgagc | SDM |
| 2159-G50A RP | gctcaccgcc**gcc**tgggcgacgt | SDM |
| 2159-W51A FP | cgcaacgtcgccgcgccggcggtgag | SDM |
| 2159-W51A RP | ctcaccgccgg**cgc**ggcgacgttgcg | SDM |
| 2159-AGWA FP | gggactgctcac**ccccgccgc**gGcgacgttgcgcga | SDM |
| 2159- AGWA RP | tcgcgcaacgtcggcgcggcgggggtgagcagtccc | SDM |

The nucleotide changes giving appropriate mutations were underlined and the restriction sites were marked in bold.
